# Survival After Massive Potassium Cyanide Ingestion Without Antidote in a Tertiary Care Setting

**DOI:** 10.7759/cureus.103265

**Published:** 2026-02-09

**Authors:** Chinnam Vishnupriya, Rachel S Kuruvila, Anagani Hrushikesh, Naveen Kumar Veerasetty, Gireesh Kumar

**Affiliations:** 1 Emergency Medicine, Amrita Hospital, Kochi, IND; 2 Preventive and Social Medicine, Jawaharlal Institute of Postgraduate Medical Education and Research, Puducherry, IND

**Keywords:** cyanide poisoning, hepatotoxicity, n-acetylcysteine, potassium cyanide, self-harm

## Abstract

Cyanide is a rapidly acting cellular toxin that blocks mitochondrial oxidative phosphorylation, causing abrupt lactic acidosis, cardiovascular collapse, and death within minutes if untreated. Survival after massive oral ingestion is rare, and published cases almost always involve the use of specific antidotes such as hydroxocobalamin or sodium nitrite with sodium thiosulfate. A 29-year-old man with emotionally unstable personality disorder/bipolar disorder, on regular psychotropic medication, ingested approximately 8 g of potassium cyanide dissolved in water in an impulsive act of self-harm. He rapidly developed multiple episodes of green, non-bloody vomiting and loose stools and was taken to a local hospital, where he was found to have elevated lactate and creatinine. In the absence of a cyanide antidote, he was started on high-flow oxygen via a non-rebreathing mask and noradrenaline and referred to a tertiary center. On arrival, he was anxious but fully conscious, with stable oxygenation, tachycardia, and mild hypotension under vasopressor support. Arterial blood gas (ABG) showed mixed respiratory alkalosis and metabolic acidosis with hyperlactatemia, hypokalemia, and hyperglycemia. Intensive supportive care included invasive hemodynamic monitoring, aggressive crystalloid resuscitation, titrated noradrenaline, broad-spectrum antibiotics, careful renal protection, correction of electrolytes, and serial blood gas monitoring. He subsequently developed marked hepatocellular injury (peak aspartate aminotransferase: 490.5 IU/L and alanine aminotransferase: 1069.4 IU/L) with preserved bilirubin and coagulation profile, for which N-acetylcysteine infusion and hepatoprotective agents were administered, with excellent response. Urine toxicology performed later in the course was negative for cyanide and thiocyanate, likely due to delayed sampling of approximately 11 hours. He was discharged on Day 13 in a stable condition with improving liver enzymes and arranged follow-up with hepatology and psychiatry. This case demonstrates that survival is possible after apparently massive potassium cyanide ingestion, even without antidote availability, when clinicians rapidly recognize the diagnosis and provide meticulous supportive care. It also highlights critical gaps in antidote access and underscores the need for integrated psychiatric follow-up and tighter control of online cyanide sales, particularly in low-resource settings.

## Introduction

Cyanide interferes with cellular respiration by binding ferric iron in mitochondrial cytochrome c oxidase, blocking electron transport and oxidative phosphorylation. Cells are compelled to undergo anaerobic metabolism, resulting in accelerated lactate production and tissue hypoxia, even in the presence of normal or elevated oxygen levels. This condition frequently culminates in death due to cardiovascular collapse or respiratory failure. The estimated lethal oral dose of potassium cyanide in adults is around 200-300 mg, though variability exists depending on purity, timing, and host factors [1-4]. Most contemporary reports of survival after acute cyanide poisoning describe early administration of specific antidotes, such as hydroxocobalamin or sodium nitrite plus sodium thiosulfate, combined with high-flow oxygen and aggressive resuscitation. However, in many EDs, especially in low- and middle-income countries, cyanide antidotes are unavailable or difficult to obtain promptly [2]. Although several reports describe cyanide inhalation and ingestion treated with antidotes, reports of survival after large oral doses managed without antidotes are rare [4-7]. This case is notable for three reasons. First, the ingested amount of approximately 8 g of potassium cyanide is far above typical lethal doses. Second, the patient was managed entirely with supportive care because no antidote was available locally. Third, he developed delayed but reversible severe transaminitis, raising important questions about cyanide-related mitochondrial and hypoxic liver injury and the potential role of N-acetylcysteine (NAC). The narrative also illustrates the human experience of a young man with chronic mental health difficulties whose impulsive act intersected with toxicology, critical care, and systemic limitations [8].

## Case presentation

A 29-year-old man from a semi-urban background was brought to the ED by his mother following intentional ingestion of potassium cyanide, which had been obtained from an online source. He was under long-term psychiatric care with a diagnosis of emotionally unstable personality disorder/bipolar disorder and was taking desvenlafaxine 100 mg at night, bupropion sustained release once daily, quetiapine 50 mg at night, valproate controlled release 300-500 mg daily, and etizolam 0.25 mg in divided doses. There was no history of cardiovascular, hepatic, or renal disease, substance misuse, or previous suicide attempts, and the family medical history was unremarkable.

On the morning of Day 0, following an intense interpersonal conflict and a subjective escalation of hopelessness, he dissolved approximately 8 g of commercially purchased potassium cyanide in about 1 L of water and consumed the mixture at around 10:00, later stating that he wanted to “end everything quickly.” The powder had been ordered online some days earlier, ostensibly for jewelry-related work, but was retained with suicidal intent [9].

Within minutes, he experienced an acrid taste, throat burning, and abdominal discomfort, progressing to repeated non-bloody, green-colored vomiting and several episodes of loose stools, accompanied by dizziness, palpitations, and a sense of impending death. Alarmed, he informed his mother, who rushed him to the nearest government medical college hospital, approximately 20 km away.

At the referring hospital, he was drowsy but arousable, with a blood pressure of 110/60 mmHg and tachycardia (129 beats/min); oxygen saturation on room air was acceptable. Point-of-care tests suggested elevated lactate (2.7 mmol/L) and creatinine (2.08 mg/dL), and clinicians suspected cyanide poisoning based on the history provided by the patient and family. High-flow oxygen via a non-rebreathing mask (NRBM) was initiated, IV crystalloids were administered, and noradrenaline infusion (10 mcg/min) was started for shock. However, the poison kit did not contain a cyanide antidote, and nearby pharmacies also lacked hydroxocobalamin or sodium thiosulfate. Recognizing the gravity of the situation, the team arranged an urgent transfer to a tertiary care center with intensive care facilities [5].

Upon arrival at the tertiary ED, at approximately 8:20 PM on the same day, the patient was anxious but fully conscious, oriented, and appropriately responsive to questions. Airway assessment showed a patent airway free of secretions or stridor; breathing revealed a respiratory rate of 20/min, equal bilateral air entry, oxygen saturation of 98% on room air (improving further on the NRBM), and no wheezes or crackles. Circulatory assessment revealed blood pressure of 110/60 mmHg on low-dose noradrenaline, heart rate of 129/min, warm peripheries, capillary refill under two seconds, and no peripheral edema. Disability assessment indicated a Glasgow Coma Scale of E4V5M6, bilaterally equal 2-mm pupils reactive to light, and no focal neurological deficits. Exposure examination found the patient afebrile, without rashes, cyanosis, oral burns, or notable odor, alongside normal heart sounds, present bowel sounds, and a soft, nontender abdomen.

**Figure 1:**
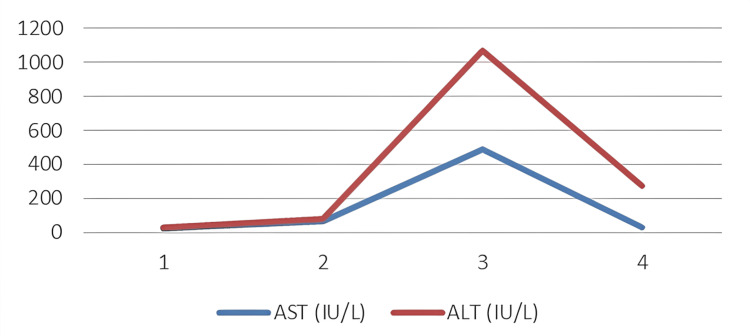
Graph depicting trends in liver enzyme levels during the hospital stay ALT, alanine aminotransferase; AST, aspartate aminotransferase

Given the history of a highly lethal ingestion and evolving systemic involvement, he was moved to a high-dependency area with continuous cardiorespiratory and pulse oximetry monitoring. Initial blood investigations, as depicted in Table 1, were normal except for a mildly elevated creatinine, suggesting acute kidney involvement. Although renal function improved over the following days, liver enzyme levels began to rise, with peak values observed on Days 7 and 8, as depicted in Figure 1. The patient remained clinically well, with no encephalopathy, coagulopathy, or jaundice, and ultrasound findings were normal. After NAC and hepatoprotective therapy, transaminases showed a steady downward trend [10], reaching values close to baseline by the time of discharge and normalizing at the next outpatient follow-up. C-reactive protein levels were elevated during the early phase and gradually declined, consistent with systemic inflammatory response and ongoing infection prophylaxis.

**Table 1:** Laboratory investigations at the tertiary care hospital ALT, alanine aminotransferase; AST, aspartate aminotransferase; INR, international normalized ratio

Test	Day 1	Day 4	Day 7-8	Day 13	Reference range
Hemoglobin (g/dL)	16.7	12.9	11.2	14.8	13.5-17.5
Total leukocyte count (K/µL)	20	3.48	3.48	6.96	4-11
Platelets (K/µL)	249	135	135	380	150-450
Urea (mg/dL)	39.5	29.6	12.2	21.5	18-55
Creatinine (mg/dL)	1.88	0.79	0.59	0.69	0.7-1.2
Sodium (mEq/L)	135.7	136.9	139.7	135.4	135-145
Potassium (mEq/L)	3.3	3.2	3	4.2	3.5-5.5
AST (IU/L)	25.7	65.3	490.5	29.2	8-48
ALT (IU/L)	28.4	76.9	1069.4	273.4	4-36
Total bilirubin (mg/dL)	0.93	0.78	0.82	0.58	0.1-1.2
INR	0.91	-	0.97	-	0.8-1.2
C-reactive protein (mg/L)	6.11	22.04	22.96	4.12	0-5

The arterial blood gas (ABG) analysis findings are shown in Table 2. The results were compatible with early cyanide poisoning in a patient who was hyperventilating and partially compensated. Serial ABGs over the ensuing hours demonstrated a gradual decline in lactate levels, correction of the acid-base disturbance, and return of renal function to baseline without the need for renal replacement therapy, with supportive care alone [3,11].

**Table 2:** ABG findings ABG, arterial blood gas; ECF, extracellular fluid

Parameter	Day 1	Day 8	Reference range
pH	7.445	7.395	7.35-7.45
PaCO₂ (mmHg)	26.1	39.6	34-45
Lactate (mmol/L)	2.7	1.4	0.6-1.4
Bicarbonate (mmol/L)	17.7	23.1	22-26
cBase (ECF) (mmol/L)	-5.7	-0.3	-2 to +2
Potassium (mmol/L)	3.4	3.7	3.4-4.5
Glucose (mg/dL)	206	163	<140

A 2D echocardiogram showed normal chamber sizes, preserved left ventricular systolic and diastolic function, only mild valvular regurgitation, and no evidence of pulmonary hypertension or pericardial effusion. Abdominal ultrasonography revealed normal liver size and echotexture, patent portal and hepatic veins, normal kidneys and spleen, and no free fluid.

The toxicology workup included a serum paracetamol level (<5 µg/mL), a multidrug panel for common antidepressants and antipsychotics (negative), and urine toxicology for cyanide, thiocyanate, and benzodiazepines, which was negative when performed several days after ingestion. The negative cyanide/thiocyanate result was interpreted cautiously, as cyanide undergoes rapid detoxification to thiocyanate and renal elimination, and delayed sampling may miss the window of detection in survivors [10,11].

Alternative diagnoses, including sepsis, other ingested poisons, and primary hepatic disease, were considered but felt to be less likely given the temporal relationship to cyanide ingestion, the characteristic early metabolic pattern, subsequent clinical improvement, and negative testing for other common toxins [12].

Management focused on aggressive supportive care, as specific antidotes were unavailable at both institutions [4]. Oxygen was administered via an NRBM at 15 L/min from the time of arrival until the end of the first critical period to maintain high dissolved oxygen levels and support residual mitochondrial function [3]. IV crystalloids (normal saline) were infused at 150 mL/h with boluses as required, and noradrenaline infusion was titrated under arterial line-guided monitoring to maintain adequate mean arterial pressure. As hemodynamic stability was achieved over the first few days, vasopressors were gradually tapered and discontinued.

An arterial line was used for continuous blood pressure monitoring and frequent blood gas assessment. Urine output, lactate levels, renal and liver function, and inflammatory markers were also closely monitored. Empirical IV ceftriaxone and doxycycline were initiated in the ED and later escalated to piperacillin-tazobactam for broader coverage, considering the risk of aspiration and gastrointestinal translocation in the setting of repeated vomiting and diarrhea. Fluid balance and creatinine levels were closely monitored, and nephrotoxic agents were avoided. Hypokalemia was corrected with IV potassium chloride infusions administered via controlled pumps, with frequent electrolyte monitoring.

With rising transaminases but preserved synthetic liver function, the hepatology team recommended NAC infusion using a modified acetaminophen protocol, along with oral hepatoprotective agents such as ursodeoxycholic acid and S-adenosyl-L-methionine. NAC was continued for five days, during which liver enzymes peaked and then steadily declined [13].

During the second week of hospitalization, the patient developed pruritic erythema over the groin, which was not attributed to cyanide toxicity. This was diagnosed as scrotal dermatitis and was managed successfully with topical antibiotic and emollient therapy. Once medically stable, he was transferred to psychiatry for a comprehensive assessment, medication review, psychoeducation, and safety planning prior to final discharge. Gastric lavage or activated charcoal was not performed at the tertiary center because several hours had elapsed since ingestion and the patient had already experienced spontaneous emesis; the risk of aspiration was judged to outweigh potential benefit [5].

Psychiatric evaluation confirmed a diagnosis of emotionally unstable personality disorder/bipolar disorder, and his psychotropic regimen was adjusted, with emphasis on regular follow-up, medication adherence, and crisis management strategies. The patient and his family elected to continue long-term psychiatric care with their local treating team, with a formal handover from the hospital psychiatrists.

At discharge on Day 13, he was afebrile, hemodynamically stable, tolerating oral intake, and ambulating independently, with no residual neurological deficits or gastrointestinal symptoms. He received clear written instructions to avoid hepatotoxic and nephrotoxic medications and herbal preparations, maintain abstinence from alcohol, and seek urgent medical attention for abdominal pain, vomiting, jaundice, confusion, or bleeding. A summary of key events from ingestion to discharge is presented in Table 3.

**Table 3:** Clinical timeline of key events during the hospital stay ABG, arterial blood gas; ALT, alanine aminotransferase; AST, aspartate aminotransferase; INR, international normalized ratio; NAC, N-acetylcysteine; NRBM, non-rebreathing mask

Days	Events
Day 0	The patient ingested approximately 8 g of potassium cyanide dissolved in water, triggering immediate vomiting and diarrhea. He presented to a local hospital where cyanide poisoning was suspected, prompting administration of high-flow oxygen via NRBM, initiation of noradrenaline, and transfer to a tertiary care center due to the absence of an antidote. That evening, evaluation at the tertiary ED revealed a mixed acid-base disturbance on ABG analysis. Ongoing management included broad-spectrum antibiotics, IV fluids, vasopressors, and placement of central venous and arterial lines.
Days 1-3	Close monitoring continued as lactate and creatinine improved, hypokalemia was corrected, and vasopressors were tapered.
Days 3-8	Transaminases rose sharply (AST/ALT ~490/1069 IU/L) despite near-normal bilirubin and INR, prompting initiation of NAC infusion and hepatoprotective therapy. Urine toxicology for cyanide/thiocyanate was negative.
Days 8-12	The patient was transferred to psychiatry and subsequently to hepatology. Liver enzymes showed a declining trend, and the patient remained clinically asymptomatic.
Day 13	The patient was discharged with arrangements for outpatient follow-up.

From the patient’s perspective, during recovery, he described intense remorse and gratitude at having survived. He reported that, in the minutes following ingestion, “everything slowed down,” and that he regretted his action almost immediately but felt trapped by the rapid onset of symptoms. He expressed surprise at surviving what he believed to be a “sure-shot” method and acknowledged underestimating the emotional impact on his family. The prolonged hospital stay, repeated blood draws, and transfers between departments made him realize “how many people were fighting” for him, which became a turning point in his willingness to engage in therapy. His mother shared feelings of guilt for not recognizing the depth of his distress, along with anger at how easily cyanide had been obtained online. She expressed hope that sharing their experience might contribute to tighter regulatory controls and help prevent similar crises in other families.

## Discussion

This case illustrates that, although cyanide is notorious for its rapid lethality, survival is possible after an apparently massive ingestion when clinicians respond promptly with meticulous supportive care. Several aspects merit discussion, including the diagnostic approach in the absence of cyanide level confirmation, the pathophysiology underlying the observed pattern of organ involvement, the role of NAC, and broader systems-level implications [5,8].

In many settings, blood cyanide levels and rapid toxicology assays are unavailable, or results are delayed beyond the window in which they can guide acute management. Diagnosis, therefore, relies on a combination of exposure history, compatible clinical features, and supportive laboratory findings, particularly severe lactic acidosis that is disproportionate to the degree of hemodynamic compromise. Our patient presented with a clear history of potassium cyanide ingestion, early gastrointestinal distress, hypotension, elevated lactate, and a mixed acid-base disturbance, all of which are consistent with acute cyanide toxicity [5,10].

The subsequent negative urine cyanide/thiocyanate test does not negate the diagnosis, as cyanide is rapidly detoxified in the liver via rhodanese-mediated conversion to thiocyanate using endogenous sulfur donors, followed by renal excretion. As a result, survivors with delayed sampling may have undetectable levels. Clinicians should therefore be cautious not to exclude cyanide poisoning solely on the basis of late negative toxicology results when the exposure history and early physiological findings are strongly suggestive [8].

The literature indicates that oral doses as low as 200-300 mg of potassium cyanide can be fatal, with many deaths occurring within minutes. If the ingested amount in this case was close to 8 g, several mitigating factors may explain survival [8,12]. Incomplete absorption is likely, as rapid and profuse vomiting and diarrhea may have expelled a substantial portion of the toxin before systemic absorption [5]. The purity of the product is also uncertain, as it was obtained from an online source, its concentration may have varied, and it may have been less pure than indicated on the label. Prompt supportive care, including early administration of high-flow oxygen, IV fluids, and vasopressors, likely limited the severity and duration of shock and tissue hypoxia. In addition, interindividual variability in endogenous detoxification capacity, including the availability of sulfur donors, may have influenced the clinical outcome. Even if the absorbed dose was lower than reported, the clinical and biochemical findings underscore that supportive measures alone can be sufficient in selected cases when instituted early and delivered effectively.

The patient’s delayed but marked transaminitis, with preserved bilirubin levels and international normalized ratio, is consistent with hypoxic hepatitis or ischemic hepatopathy, in which transient hepatic hypoxia leads to hepatocellular necrosis, with recovery expected once perfusion is restored. Cyanide’s direct mitochondrial toxicity may further impair hepatocellular oxidative phosphorylation, increasing susceptibility to liver injury [12,13]. NAC has antioxidant, vasodilatory, and microcirculatory effects beyond its established role in paracetamol overdose and has demonstrated benefit in some cases of nonparacetamol acute liver failure. Although causality cannot be definitively established, the temporal association between NAC initiation and improvement in transaminase levels, together with its favorable safety profile, supports its pragmatic use in similar cases of toxin-related or hypoxic liver injury.

From a mental health perspective, this case highlights the vulnerability of individuals with personality and mood disorders to impulsive, high-lethality self-harm. Long-term prevention requires robust psychiatric follow-up, evidence-based pharmacotherapy, psychotherapies targeting emotion regulation and interpersonal functioning, and active family involvement [9].

At a systems level, this case exposes critical gaps in antidote availability and the regulation of hazardous substances. International guidelines recommend that EDs at risk of cyanide exposure maintain adequate stocks of cyanide antidotes and establish protocols for empiric treatment in suspected cases. Concurrently, the ease of online access to cyanide, ostensibly for industrial or laboratory use, raises significant ethical and regulatory concerns. Strengthened restrictions, purchaser verification mechanisms, and enhanced public health oversight are needed to reduce the risk of misuse for self-harm [5,7]. In the United States, the Food and Drug Administration has approved cyanide antidote products, including hydroxocobalamin injection, sodium nitrite injection, and sodium thiosulfate injection [14].

## Conclusions

Survival is possible after apparently massive potassium cyanide ingestion, even in the absence of an antidote (although systemic cyanide levels were not established), when clinicians provide rapid, high-quality supportive care, including high-flow oxygen, hemodynamic stabilization, and meticulous correction of metabolic derangements. In resource-limited settings, a clear exposure history combined with hyperlactatemia and characteristic ABG abnormalities should prompt empiric management for cyanide poisoning without waiting for confirmatory toxin levels, which may be unavailable or falsely negative when sampling is delayed. Cyanide poisoning can be associated with delayed but reversible hepatocellular injury resembling hypoxic hepatitis; NAC may be a reasonable adjunctive therapy given its favorable safety profile and potential benefits in non-paracetamol acute liver injury. Comprehensive psychiatric assessment, ongoing mental health care, and family involvement are essential following high-lethality self-harm, while health systems should improve access to cyanide antidotes and strengthen regulation of online cyanide sales to prevent similar attempts.
